# Transformation of Classical Hodgkin Lymphoma Into Non-Hodgkin Lymphoma: Three Case Reports From the West Bank

**DOI:** 10.1155/crom/4802098

**Published:** 2025-10-29

**Authors:** Mohammed B. Abboushi, Omar Marouf, Mohammed AbuBaha, Hossam Salameh, Ro'ya Soradi, Riad Ahmad Amer

**Affiliations:** ^1^Department of Internal Medicine, An-Najah National University Hospital, Nablus, State of Palestine; ^2^Department of Internal Medicine, Palestine Medical Complex, Ramallah, State of Palestine; ^3^Department of Medicine, An-Najah National University, Nablus, State of Palestine; ^4^Pathology Department, An-Najah National University Hospital, Nablus, State of Palestine; ^5^Oncology and Hematology Department, An-Najah National University Hospital, Nablus, State of Palestine

## Abstract

**Background:**

Classical Hodgkin lymphoma (cHL) is a highly curable B-cell malignancy; though, rarely, it can transform into non-Hodgkin lymphoma (NHL), including diffuse large B-cell lymphoma (DLBCL), follicular lymphoma, or marginal zone lymphoma. Reports of such transformations remain limited in the literature.

**Objective:**

The aim of this study is to describe the clinical course, histopathological findings, and outcomes of three patients with cHL who developed secondary NHL.

**Results:**

Case 1 involved transformation from cHL to CD20+/CD30+ DLBCL following multiple lines of chemotherapy and autologous stem cell transplant, eventually resulting in progressive disease and death. Case 2 transformed into follicular lymphoma Grade 3a more than a year after cHL remission, with marrow infiltration managed conservatively pending systemic therapy. Case 3 presented with composite lymphoma at diagnosis (cHL and extranodal marginal zone lymphoma) and experienced indolent but recurrent disease involving the liver, requiring multiple rounds of chemoimmunotherapy.

**Conclusion:**

Transformation of cHL into NHL, though rare, may occur years after initial treatment or concurrently at presentation. These cases underscore the importance of repeat biopsy in suspected relapses and highlight the clinical and pathological heterogeneity of such transformations. Our paper adds to the limited literature on this phenomenon and is the first of its kind reported from Palestine.

## 1. Introduction

Hodgkin's disease or Hodgkin lymphoma (HL), first designated by the British pathologist Thomas Hodgkin in 1832 [1], is a rare lymphoid tumor arising from B-cells, with a bimodal age distribution, that typically arises in cervical lymph nodes (LNs) and later spreads to axillary and inguinal LNs [2]. HL is divided into two types, which have been reported, though very rarely, to transform into other types of lymphomas, particularly diffuse large B-cell lymphoma (DLBCL), which are not well-documented.

Herein, we present cases of three patients who were first diagnosed with classical Hodgkin lymphoma (cHL), which later appeared to have transformed into DLBCL, which is considered to be very rare, with limited data on such occurrences. We describe their clinical course, treatment, and outcome in a report that, to our knowledge, is the first of its kind in the West Bank of Palestine.

## 2. Case Reports

### 2.1. Case 1

A 61-year-old male with a 10-year history of Type 2 diabetes mellitus presented in 2021 with generalized pruritus unresponsive to antihistamines, followed by right inguinal lymphadenopathy. Excisional biopsy confirmed cHL, Stage IIIA. He received two cycles of ABVD (adriamycin, bleomycin, vinblastine, dacarbazine) in August 2021, achieving a complete metabolic response on PET/CT.

By mid-2022, imaging demonstrated disease relapse. He was treated with four cycles of ICE (ifosfamide, carboplatin, etoposide), achieving remission on PET/CT. He subsequently underwent successful stem cell mobilization with high-dose cyclophosphamide and filgrastim, followed by autologous stem cell collection in preparation for autologous bone marrow transplantation (auto-BMT).

In August 2022, he underwent conditioning with the LEAM protocol (lomustine, etoposide, cytarabine, melphalan), followed by autologous stem cell reinfusion. His course was complicated by febrile neutropenia due to *Escherichia coli* bacteremia, managed with piperacillin–tazobactam and amikacin. He also developed severe thrombocytopenia requiring multiple platelet transfusions. He subsequently recovered and was discharged.

**Figure 1:**
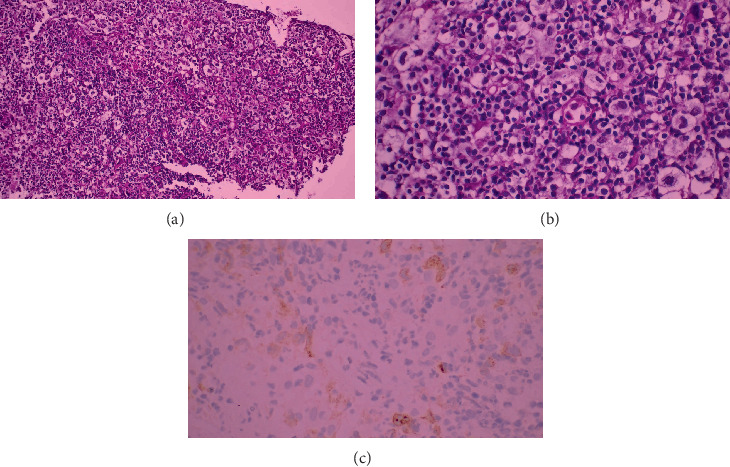
First case demonstrating polymorphous population of cells includes large cells with (a, b) prominent nucleoli and abundant eosinophilic cytoplasm and (c) CD30 immunostaining.

Follow-up PET/CT in early 2023 revealed cervical and retroperitoneal lymphadenopathy. Biopsy of a left cervical LN demonstrated transformation to DLBCL (Figure 1), positive for CD20 and CD30, with a Ki-67 proliferation index of 95%. Bone marrow biopsies showed no infiltration. He received five cycles of R-DHAP (rituximab, dexamethasone, cytarabine, cisplatin), completed in August 2023. PET/CT in October showed persistent supraclavicular disease with high SUV uptake. Repeat excision confirmed CD30-positive DLBCL.

Despite two cycles of brentuximab vedotin initiated in October 2023, disease progressed. CT in January 2024 demonstrated widespread lymphadenopathy, pulmonary nodules, and a renal lesion. The patient declined further intensive regimens, including hyper-CVAD and rituximab, and opted for supportive care.

In February 2024, he was hospitalized with neutropenic sepsis. He presented with hypoxemia, hypotension, and bilateral pulmonary infiltrates. Nasopharyngeal swab was positive for influenza A, and blood cultures grew gram-negative bacteria. Despite broad-spectrum antibiotics and supportive therapy, he developed septic shock and multiorgan dysfunction. He experienced cardiac arrest on February 5, 2024, and resuscitation was unsuccessful. The immediate cause of death was neutropenic septic shock secondary to pneumonia and bacteremia.

### 2.2. Case 2

**Figure 2:**
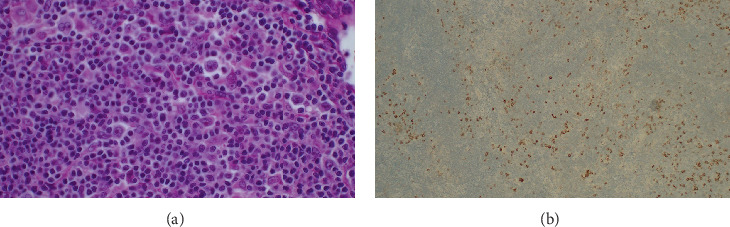
Second case demonstrating effaced lymph node with infiltration by (a) RS cells and CD30-positive RS cells.

A 67-year-old male with a history of traumatic splenectomy at age 10 presented in early 2021 with right inguinal lymphadenopathy and a thyroid nodule. LN biopsy (Figure 2) suggested follicular neoplasia in situ. A repeat excisional biopsy showed only reactive changes. CT revealed intra-abdominal and para-aortic lymphadenopathy, and intra-abdominal node biopsy confirmed cHL (nodular sclerosis subtype), Stage IIB. He completed six cycles of ABVD by December 2021. Bone marrow biopsy at baseline showed no involvement.

Restaging PET/CT in January 2022 revealed residual mesenteric lymphadenopathy. By April 2022, repeat axillary node biopsy showed no lymphoma, and the patient remained asymptomatic. In January 2023, surveillance PET/CT demonstrated progressive lymphadenopathy. Cervical node biopsy confirmed follicular lymphoma (FL) Grade 3a. FISH analysis supported the diagnosis.

Given the absence of symptoms and low tumor burden, the patient was initially managed with watchful waiting. However, by June 2023, cervical, axillary, and inguinal lymphadenopathy had progressed. Biopsies revealed FL Grade 1. PET/CT in November 2023 showed further progression, and a right axillary biopsy in December confirmed recurrent FL Grade 3a with a follicular growth pattern.

In March 2024, the patient developed fatigue and anemia (hemoglobin decline from 11.6 to 9.3 g/dL). Bone marrow biopsy showed 70% involvement by low-grade FL with paratrabecular distribution, consistent with marrow infiltration, although CD10 and BCL6 were negative. Concurrently, he developed atrial fibrillation with a rapid ventricular response. Echocardiogram revealed biatrial enlargement, moderate mitral regurgitation, and mild aortic stenosis.

After stabilization with bisoprolol and rivaroxaban, systemic treatment was indicated due to disease burden and marrow involvement. Bendamustine plus rituximab was selected as the planned regimen, pending full cardiac stabilization.

### 2.3. Case 3

A 57-year-old male with no significant medical history presented in late 2020 with 10 months of progressive right cervical lymphadenopathy, associated with night sweats and intermittent fever. Excisional biopsy confirmed cHL (nodular sclerosis subtype). Bone marrow biopsy in December 2020 showed trilineage hematopoiesis without involvement. He received one cycle of ABVD in January 2021.

Staging CT revealed hepatic lesions. Liver biopsy demonstrated low-grade B-cell lymphoma consistent with extranodal marginal zone lymphoma, establishing a diagnosis of composite lymphoma (cHL with marginal zone lymphoma). He received five cycles of R-CHOP and neck radiotherapy. Follow-up PET/CT showed persistent hepatic lesions, and repeat biopsy confirmed low-grade B-cell lymphoma.

**Figure 3:**
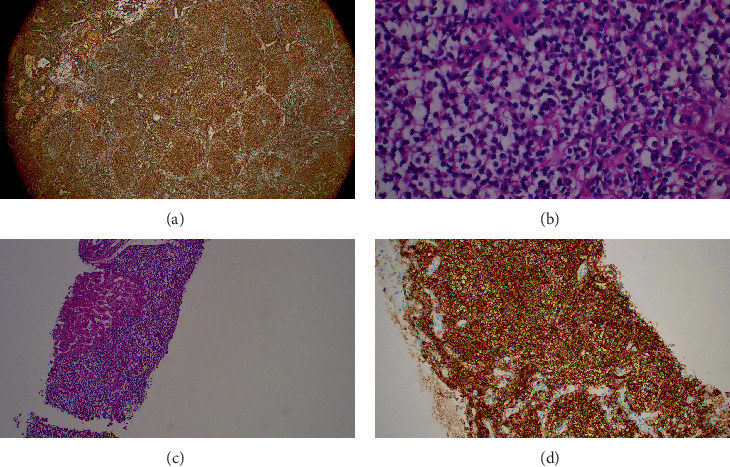
(a–c) Third cases demonstrating sections of hepatic tissue heavily infiltrated by small lymphocytes with monocytoid appearance. (d) CD20 immunostaining is diffusely positive.

In early 2023, surveillance imaging revealed progression of liver disease. Repeat liver biopsy again showed low-grade B-cell lymphoma without Reed–Sternberg cells (Figure 3). Given stable liver enzymes and the absence of systemic symptoms, a watch-and-wait strategy was adopted.

By January 2024, PET/CT showed increased SUV and enlargement of hepatic lesions. Multidisciplinary consensus recommended bendamustine–rituximab. Bone marrow biopsy in February 2024 revealed normal hematopoiesis without infiltration, though hemophagocytosis was noted. The patient received four cycles of bendamustine–rituximab, completed in May 2024.

Restaging CT revealed a new left hepatic lesion with peripheral and central enhancement, while previously known hepatic and adrenal lesions remained stable. Pulmonary nodules also remained unchanged. Given the absence of new systemic symptoms, close radiologic surveillance was recommended with consideration for dedicated liver protocol imaging.

This case illustrates the complex course of composite lymphoma, with overlapping histologies and divergent clinical trajectories. Despite multiple relapses, the patient maintained a favorable performance status and responded to therapy, highlighting the challenges of managing such rare presentations.

**Figure 4:**
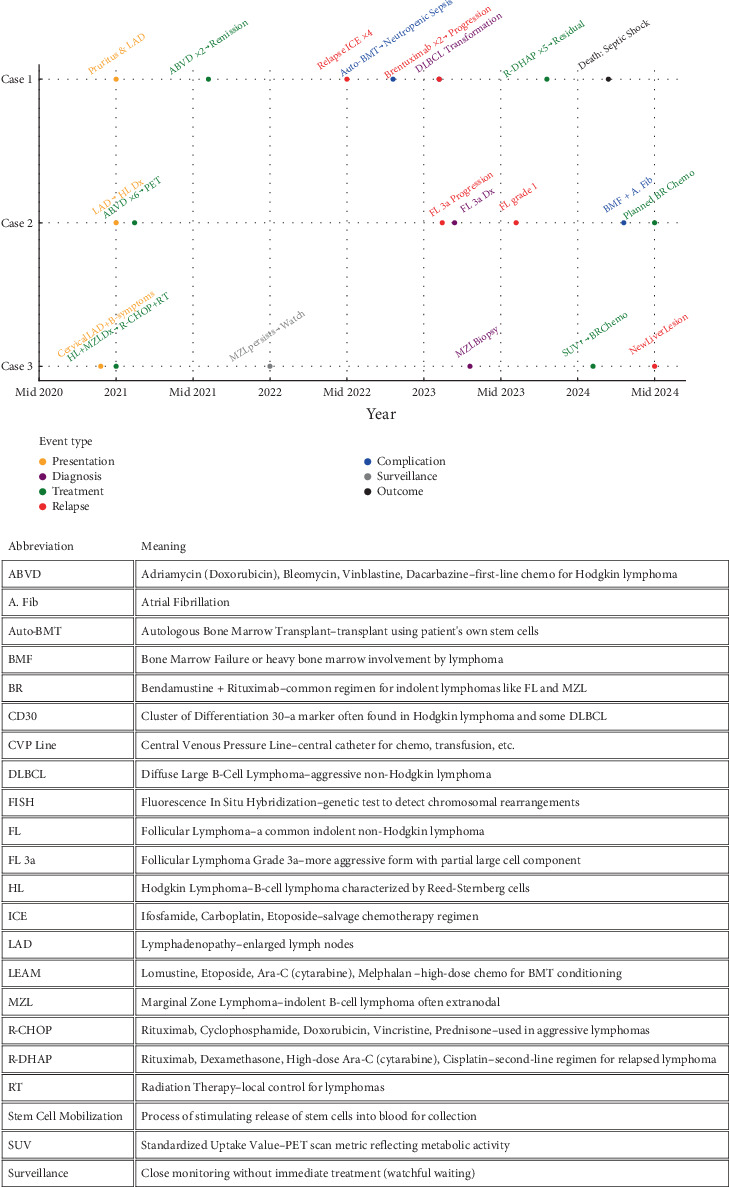
Chronological timeline of the course for our series of patients.

Chronological timeline of the course of the three patients is demonstrated in Figure 4 and Table 1.

## 3. Discussion

HL is a B-cell–derived malignancy characterized by a bimodal age distribution and generally favorable outcomes, with long-term remission rates approaching 80% in modern series. Secondary malignancies after successful treatment are uncommon, occurring in approximately 1.5% of patients, as demonstrated in a large cohort of 11,841 cases reported by Eichenauer et al. [3]. Although rare, transformation of cHL into non-Hodgkin lymphoma (NHL) represents a clinically significant event that can alter prognosis and management [3], and features and patient characteristics are demonstrated in Table 2.

Transformation patterns vary across histologic subtypes. Reported frequencies include FL (30%–40%), nodular lymphocyte–predominant HL (10%–30%), lymphoblastic leukemia (5%–10%), small lymphocytic lymphoma/chronic lymphocytic leukemia (≈5%), and marginal zone lymphoma (≈4%) [4].

The mechanisms underlying transformation remain incompletely understood. Hypothesized contributors include treatment-induced immunosuppression and Epstein–Barr virus (EBV) positivity [3, 4]. Other risk factors associated with transformation include advanced age and elevated lactate dehydrogenase (LDH) levels [3]. In addition, Küppers et al. demonstrated that germline polymorphisms may predispose certain individuals to the development of multiple lymphoid malignancies concurrently [5]. Although positive EBV status is associated with lymphoma development, this was not assessed in our patients.

From a clinical perspective, transformation tends to occur in older patients and may mimic recurrent HL. Therefore, NHL should be carefully excluded in any patient with apparent HL relapse, particularly when the clinical course or histology appears atypical [3]. In alignment with this, the HL committee of the Lymphoma Study Association (LYSA) recommends repeat biopsy at the time of relapse, especially if it occurs more than 1 year after initial treatment, to rule out transformation [6].

The time course of transformation is also relevant. In a cohort of 164 patients, Virga et al. observed that three individuals developed NHL at least 18 months after achieving complete remission for cHL [7].

This timeline is consistent with our patients, all of whom experienced transformation more than 1 year after initial therapy. Regarding outcomes, Smith et al., in a series of 214 patients, reported cure rates of up to 55% when second-line chemotherapy was followed by high-dose therapy and autologous stem cell transplantation. Adverse prognostic factors included bulky disease (≥ 5 cm) and the presence of multiple risk features [8].

## 4. Conclusion

Transformation of cHL into NHL is an uncommon but clinically significant event that may complicate diagnosis and management. Our report highlights both metachronous and synchronous transformations, with variable histological subtypes and treatment responses. Timely rebiopsy of recurrent or progressive disease remains essential to guide accurate diagnosis and appropriate therapy. Greater awareness and reporting of such transformations are necessary to improve prognostication and optimize management strategies for affected patients.

## Figures and Tables

**Table 1:** Summary of clinical course and manifestations in our three patients.

**Case**	**Age**	**Gender**	**Initial presentation**	**Treatment course**	**Transformation**	**Final course**	**Key outcome markers**
1	61	M	Generalized pruritus → right inguinal LAD	- ABVD ×2 → remission (2021) - Relapse → ICE ×4 (2022) - Auto-BMT with LEAM (Aug 2022) - R-DHAP ×5 (2023) - Brentuximab ×2 (2023)	To CD30 + DLBCL (early 2023)	Died Feb 2024 from neutropenic septic shock	- Ki-67: 95% in transformed DLBCL - CD20/CD30 positive - Bone marrow: Negative - PET/CT: SUV high in supraclavicular nodes (Oct 2023) - Severe thrombocytopenia during BMT
2	67	M	Right inguinal LAD + thyroid nodule	- ABVD ×6 (2021) - FL 3a (Feb 2023) - FL Grade 1 (Jun 2023) - FL 3a again (Dec 2023) - Planned BR chemo (2024)	To FL Grade 3a (Feb 2023)	Planned BR chemo pending cardiac clearance	- Bone marrow: 70% involvement (Mar 2024) - CD10/BCL6 negative - Hb: Dropped from 11.6 → 9.3 - ECHO: Biatrial dilation, moderate MR, mild AS - PET/CT: Progression on imaging (Nov 2023)
3	57	M	Right cervical LAD + B-symptoms	- 1 cycle ABVD (2021) - Composite lymphoma (HL + MZL) - R-CHOP ×5 + RT - Surveillance until 2024 - BR chemo ×4 (May 2024)	Composite lymphoma at diagnosis (2021)	Ongoing surveillance after partial response	- Liver biopsy: Persistent MZL without RS cells - Bone marrow: No infiltration, showed hemophagocytosis - PET/CT: SUV ↑ (Jan 2024) - New liver lesion with central/peripheral enhancement (May 2024) - Stable lung nodules

*Note:* CD20/CD30, B-cell/tumor markers; Ki-67, proliferation index (% of cells dividing); > 80%, aggressive disease; higher, more active disease.

Abbreviation: SUV, standardized uptake value (from PET/CT).

**Table 2:** Course and clinical manifestations of 11,841 patients with cHL 106 months after first-line treatment.

Rate of transformation	175 patients had developed non-Hodgkin lymphoma (NHL) (1.5%)		
Sex	Male (63.4%)		Female (36.6%)
Type of transformation in 170 of them	Indolent B-cell NHL (20.5%)	Aggressive B-cell NHL (60%)	T-cell NHL (19.4%)

## Data Availability

The data that support the findings of this study are available on request from the corresponding author. The data are not publicly available due to privacy or ethical restrictions.
