# Correction to “Transformation of Classical Hodgkin Lymphoma Into Non‐Hodgkin Lymphoma: Three Case Reports From the West Bank”

**DOI:** 10.1155/crom/9827843

**Published:** 2026-05-30

M. B. Abboushi, O. Marouf, M. AbuBaha, et al., “Transformation of Classical Hodgkin Lymphoma Into Non‐Hodgkin Lymphoma: Three Case Reports From the West Bank”, *Case Reports in Oncological Medicine* 2025 (2025): 4802098, 10.1155/crom/4802098

In the article titled “Transformation of Classical Hodgkin Lymphoma Into Non‐Hodgkin Lymphoma: Three Case Reports From the West Bank,” the corresponding author details for Hossam Salameh were incorrect. The correct corresponding information is shown below:

Correspondence should be addressed to Riad Ahmad Amer (riadamer@najah.edu).

We apologize for this error.

